# A Theoretical Investigation of Third-Order Optical Susceptibility in Metronidazolium-Picrate Crystal and Its Potential for Quantum Memory Applications

**DOI:** 10.1021/acsomega.5c03825

**Published:** 2025-08-21

**Authors:** Clodoaldo Valverde

**Affiliations:** 1 Laboratório de Modelagem Molecular Aplicada e Simulação (LaMMAS), 271384 Universidade Estadual de Goiás, Anápolis, GO 75001-970, Brazil; 2 Universidade Paulista − UNIP, Goiânia, GO 74845-090, Brazil

## Abstract

In this work, we report a theoretical investigation of the third-order nonlinear optical properties of the metronidazolium-picrate salt. The effects of the crystal environment are accounted for by the Iterative Charge Embedding approach, and the electronic calculations are carried out at the DFT (CAM-B3LYP/6-311++G­(d,p)) level. Furthermore, we use the *ab initio* results to parametrize a cavity Quantum Electrodynamics model for a quantum memory based on the Off-Resonant Cascaded Absorption protocol. The system’s performance is then simulated via a Lindblad-type master equation that includes realistic decoherence channels. Our results confirm a strong third-order susceptibility (χ^(3)^) of 3.4 × 10^–20^ (m/V)^2^ at 532 nm driven by significant charge polarization in the crystal. The quantum memory simulations, initiated with a single-photon Fock state, reveal that protocol fidelity is critically dependent on the cavity quality factor. A peak retrieval fidelity of 84.51% is achieved in the strong coupling regime, which collapses to less than 1% when the system leaves this regime. These findings demonstrate that METPA is a promising material for quantum photonics, where its strong intrinsic electronic properties can be harnessed in engineered cavity Quantum Electrodynamics systems to realize high-fidelity quantum information protocols.

## Graphical abstract

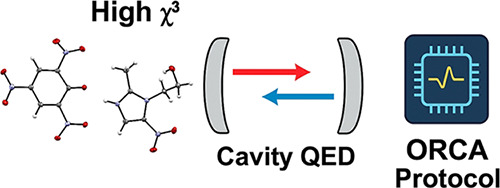

## 1 Introduction

Nonlinear optics has garnered significant attention due to its crucial role in advancing quantum technologies, including quantum memories^1,2^ and optoelectronic devices^3,4^ designed for secure communication and information processing.^5^ The effectiveness of these technologies depends directly on the ability of materials to exhibit high values of nonlinear susceptibility, particularly of third-order (χ^(3)^),^6,7^ which is closely related to second molecular hyperpolarizability and asymmetric electronic distribution in crystalline materials.

However, crystalline organic materials presenting high and stable values of χ^(3)^ remain scarce, which makes their practical use in compact quantum-optical systems.

The metronidazolium-picrate (METPA) salt, an organic compound recently synthesized and structurally characterized,^8^ consists of the ionic pair C_6_H_10_N_3_O_3_^+^·C_6_H_2_N_3_O_7_^–^, emerges as a potential candidate for nonlinear optical applications due to its triclinic crystalline structure that has strong intermolecular interactions, such as N–H···O and O–H···O hydrogen bonds.^8^

These interactions give thermodynamic stability and electronic anisotropy to the crystal structure of METPA, essential factors for achieving significant nonlinear optical response.

Within this context, it becomes essential to understand the effect of the crystalline environment on the nonlinear optical response of METPA. For this purpose, we constructed a bulk structure containing 548,720 atoms using the Iterative Charge Embedding (ICE) approach, an electrostatic iterative technique effective for simulating complex crystalline environments and accurately representing molecular charge distributions, often yielding results in good agreement with experimental data.^9–12^

Nonlinear optical properties of METPA, such as total dipole moment, average linear polarizability, and average second hyperpolarizability, were calculated at DFT/CAM-B3LYP level with the 6–311++G­(d,p) basis set. This methodology was previously applied successfully to organic crystalline solids, accurately predicting electronic properties,^13^ thus providing a reliable basis for estimating third-order nonlinear susceptibility (χ^(3)^).

Recent advances in quantum memories employing optimized cavities, such as the Off-Resonant Cascaded Absorption (ORCA) protocol demonstrated in room-temperature atomic vapors,^14^ highlight the importance of enhancing nonlinear light-matter interactions for scalable architectures.

Using an integrated approach that combines electrostatic modeling of the crystalline environment via the ICE method with high-level DFT calculations, we first obtained a reliable estimate of the third-order nonlinear susceptibility (χ^(3)^) for the METPA crystal. Building on this finding of a strong nonlinear response, this study then investigates the material’s potential for quantum information applications.

To this end, we simulate the performance of METPA as the active medium in an advanced quantum memory protocol, namely ORCA. This protocol was chosen as it is an intrinsically noise-free technique designed for high-fidelity quantum state storage, making it an ideal benchmark for a new material.^15^ The system’s dynamics are simulated via a Lindblad master equation that includes a comprehensive set of realistic decoherence channels, such as photonic loss, atomic decay, and pure dephasing.

Storage fidelity and average photon number will be monitored over time to assess METPA’s viability as a functional platform for quantum information technologies. This analysis bridges the electronic structure of the material and its practical applicability in third-order nonlinear optics, focusing on operations under realistic decoherence conditions.

## 2 Methods

### 2.1 Synthesis and Crystallization of METPA

The metronidazolium picrate (METPA) salt was prepared using a slow evaporation method. Equal amounts of metronidazole and picric acid were mixed in methanol at room temperature. The solution was stirred for 1 h and then filtered.

After a few days at ambient conditions, yellow crystals were formed by natural evaporation of the solvent. These crystals were used for structure determination. No further purification was applied.

### 2.2 Structural Data from Literature

The crystal structure of METPA was previously reported using single-crystal X-ray diffraction.^8^ The compound crystallizes in the triclinic system, with space group *P-*1. The reported unit cell parameters are *a* = 8.2446 Å, *b* = 9.1843 Å, *c* = 11.5832 Å, α = 106.190°, β = 99.483°, γ = 106.635°, and volume ∀ = 778.02 Å^3^, and Λ = 2 is number of molecules in the unit cell. These structural data were used as the starting geometry for all theoretical simulations in this work.

### 2.3 Crystalline Environment Simulation

To include for the polarization effects of the crystalline environment on the asymmetric unit of METPA (Figure 1), we used the Iterative Charge Embedding (ICE) approach. This approach considers long-range electrostatic interactions by embedding a single molecule within a large cluster of surrounding molecules, where each atom of these neighboring molecules is substituted by atomic point charges.

**Figure 1:**
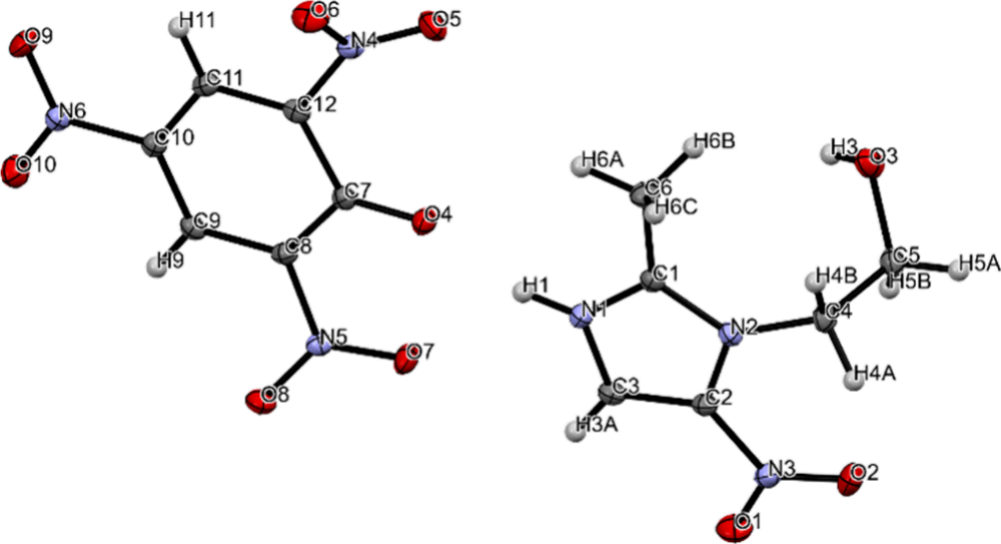
Molecule METPA.

The bulk model was constructed by replicating the unit cell in a 19 × 19 × 19 grid, generating a total of 548,720 atoms (Figure 2a), which provides a realistic electrostatic environment around the isolated molecule.

**Figure 2:**
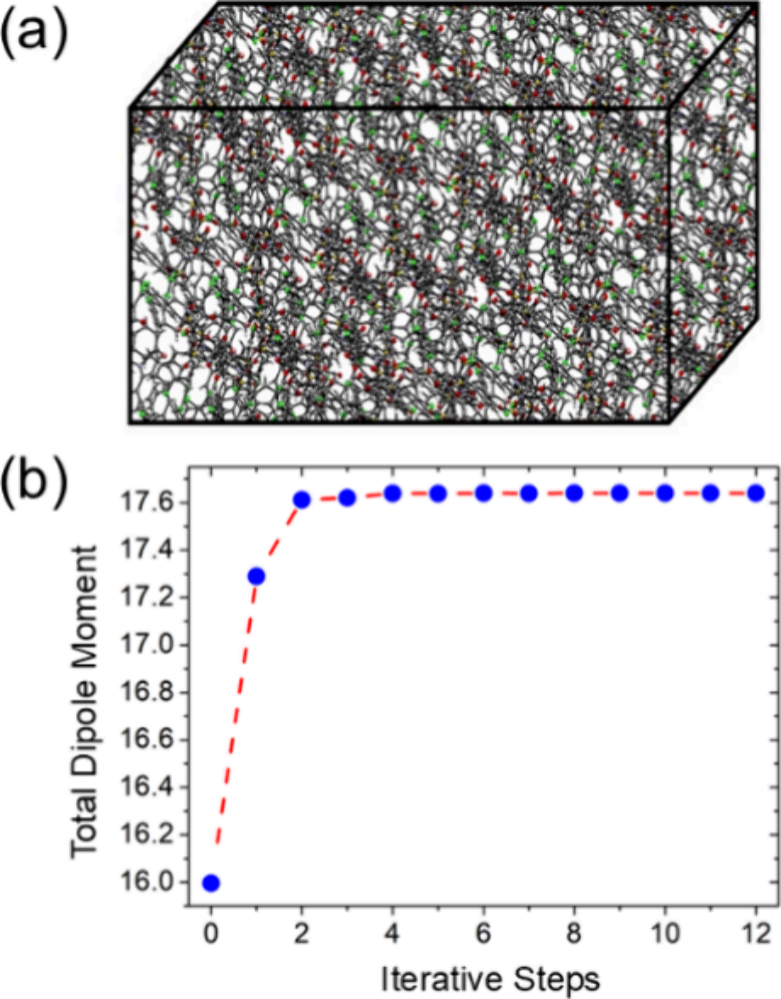
a) The bulk; b) The total dipole moment as a function of iterative steps.

The simulation starts by calculating the atomic charges for the isolated molecule using the ChelpG method. Based on these charges, a crystal-like environment is built, where all atoms of the neighbor molecules are substituted by their respective charge as found by the ChelpG method. In each ICE iteration, new charges are calculated and are substituted at the atoms of the neighbor molecules, and the procedure repeats.

This iterative process continues until the total dipole moment of the molecule becomes stable. In our simulation, the convergence was reached after several cycles, resulting in a final dipole moment of 17.64 D, see Figure 2b.

This confirms that the embedded molecule reached an electrostatically consistent state within the crystalline bulk, allowing us to proceed with the calculation of nonlinear optical properties under conditions that are more close to the crystal.

### 2.4 Electro-optical Parameters

The total dipole moment (μ), the mean linear polarizability (⟨α⟩), and the linear refractive index were determined based on the corresponding theoretical expressions,

$$\mu ={\left(\mu_{x}^{2}+\mu_{y}^{2}+\mu_{z}^{2}\right)}^{1/2}$$ 1

$$\left\langle \alpha \left(-\omega ;\omega \right)\right\rangle =\frac{1}{3}\underset{i=x,y,z}{\sum }\alpha_{ii}\left(-\omega ;\omega \right)$$ 2

$$\frac{n{\left(\omega \right)}^{2}-1}{n{\left(\omega \right)}^{2}+2}=\frac{4\pi \Lambda }{3\forall }\left\langle \alpha \left(-\omega ;\omega \right)\right\rangle$$ 3

The Clausius-Mossotti relation,^16^ as expressed in eq 3, establishes a connection between the average linear polarizability ⟨α­(−ω; ω)⟩ and the linear refractive index n­(ω) of the crystal.

The average second hyperpolarizability was calculated using equation,

$$\left\langle \gamma \left(-\omega ;\omega ;0;0\right)\right\rangle =\frac{1}{15}\underset{i,j=x,y,z}{\sum }\left(\gamma_{iijj}+\gamma_{ijij}+\gamma_{ijji}\right)$$ 4

As optical dispersion effects were not included in the present calculation, the static average second hyperpolarizability was estimated using the Kleinman symmetry approximation,^17^

$$\left\langle \gamma \left(-\omega ;\omega ;0;0\right)\right\rangle =\frac{1}{5}\left[\gamma_{xxxx}+\gamma_{yyyy}+\gamma_{zzzz}+\left(\gamma_{xxyy}+\gamma_{xxzz}+\gamma_{yyzz}\right)\right]$$ 5

The third-order nonlinear susceptibility χ^(3)^(−ω; ω; ω; – ω), related to the intensity-dependent refractive index (IDRI), can be calculated from the average second hyperpolarizability using the relation:

$$\chi^{\left(3\right)}\left(-\omega ;\omega ;\omega ;-\omega \right)=f{\left(\omega \right)}^{4}\frac{\Lambda }{\forall }\left\langle \gamma \left(-\omega ;\omega ;\omega ;-\omega \right)\right\rangle$$ 6

In this eq 6, $f\left(\omega \right)=\left(\frac{\left(n{\left(\omega \right)}^{2}+2\right)}{3}\right)$ is the Lorentz local-field factor; *n*(ω) is the refractive index, Λ is the number of molecules per unit cell and ∀ is the unit-cell volume, and ⟨γ­(−ω; ω; ω; –ω)⟩ the IDRI second hyperpolarizability was obtained using equation,

⟨γ(−ω;ω,ω,−ω)⟩≅2⟨γ(−ω;ω,0,0)⟩−⟨γ(0;0,0,0)⟩ 7

All computations were carried out at the DFT/CAM-B3LYP level with the 6–311++G­(d,p) basis set using the Gaussian 16 software package.^18^

### 2.5 Simulation of Quantum Memory Performance

In this study, we make a computational method for the simulation of ORCA quantum memory protocol. The start point is molecular properties from ab initio calculation. Using the Gaussian 16 program, the energies of 20 excited states, see Table S1, and also the complete matrix of transition dipole moments (μ_*ij*_) is calculated, see Table S2, with the TD-DFT method at CAM-B3LYP/aug-cc-pVTZ theory level, see all details in the Supporting Information. Then, with this data we can construct the Hamiltonian for a three-level system model of our molecule.

Finally, a simulation of the quantum dynamic for this system is done. This simulation includes the signal and control light fields, and the purpose is to check the fidelity for storage and for retrieval of the quantum state in the ORCA protocol.

#### 2.5.1 System Hamiltonian for the ORCA Protocol

For our system, we write the Hamiltonian in the rotating frame. This is the effective Hamiltonian that controls the coherent dynamics of the system:

$$Ĥ=\Delta_{e}{\mathrm{\sigma ̂}}_{ee}+\left(\Delta_{e}+\Delta_{f}\right){\mathrm{\sigma ̂}}_{ff}+g\left({â}^{†}{\mathrm{\sigma ̂}}_{ge}+â{\mathrm{\sigma ̂}}_{eg}\right)+\Omega \left(t\right)\left({\mathrm{\sigma ̂}}_{ef}+{\mathrm{\sigma ̂}}_{fe}\right)$$ 8

Here, the operators and terms have this meaning:

- *σ̂*_μυ_ = |μ⟩⟨υ|: These are the atomic operators for the three-level atom (|*g*⟩, |*e*⟩, |*f*⟩). For example, *σ̂*_*ge*_ is the lowering operator from state |*e*⟩ to |*g*⟩.
- *â* ^†^ and *â:* These are the bosonic creation and annihilation operators for the photon field mode inside the optical cavity.
- Δ*_e_* and Δ*_f_*: These are the detuning parameters. They describe the energy difference of the atomic levels from the frequencies of the light fields.
- g and Ω­(t): These are the coupling strengths, which we explain below.

Here is what each term of the Hamiltonian represents:

- Δ_*e*_ *σ̂*_*ee*_: The energy shift (detuning) of the intermediate state |e⟩; it sets the reference energy of |e⟩ in the rotating frame.
- (Δ*_e_* + Δ*_f_*)*σ̂*_*ff*_: The energy shift of the storage state |f⟩; the sum Δ*_e_* + Δ*_f_* ensures the correct detuning of |f⟩ relative to the control field.
- *g*(*â* ^†^ *σ̂*_*ge*_ + *âσ̂*_*eg*_): The atom–field (Jaynes–Cummings) coupling with strength g, enabling coherent exchange of excitation between |g⟩ and |e⟩ via photon creation/annihilation.
- Ω­(*t*)­(*σ̂*_*ef*_ + *σ̂*_*fe*_):The classical control-field coupling Ω­(t), driving the |e⟩↔|f⟩ transition to “write” and “read” the excitation in the ORCA protocol.

The general form of the atom-cavity coupling strength g is,

$$g=\frac{\mu_{ge}}{\hbar }\sqrt{\frac{\hbar \omega_{ge}}{2\epsilon_{0}V_{cav}}}$$ 9

where μ_*ge*_ is the transition dipole moment for the |g⟩→|e⟩ transition (the value we get from *ab initio* calculations), *V*_*cav*_ is the effective volume of the cavity mode, ω_*ge*_ is the transition frequency, and ϵ_0_ is the vacuum permittivity.

Also, the term for the control laser coupling, which is the peak Rabi frequency Ω­(t). This function is made of a sequence of two hyperbolic secant (sech) pulses, a ″write″ pulse for storing and a ″read″ pulse for retrieving the quantum state:

$$\Omega \left(t\right)=\Omega_{write}\left(t\right)+\Omega_{read}\left(t\right)$$ 10

where each individual pulse has the form:

$$\Omega_{write}\left(t\right)=\Omega_{0}\mathrm{sech}\left(\frac{t-t_{0,write}}{\tau }\right)$$ 11

and

$$\Omega_{read}\left(t\right)=\Omega_{0}\mathrm{sech}\left(\frac{t-t_{0,read}}{\tau }\right)$$ 12

The peak Rabi frequency Ω_0_ for these pulses, which defines their maximum amplitude, has the form,

$$\Omega_{0}\left(t\right)=\frac{\mu_{ef}E_{0}}{\hbar }$$ 13

where μ_*ef*_ is the transition dipole moment for the |e⟩→|f⟩ transition and *E*_0_ is the peak electric field amplitude of the control laser pulse. This two-pulse sequence allows for the coherent transfer of the excitation to the storage state |f⟩ at time *t*_0, *write*_, and its subsequent retrieval at time *t*_0, *read*_ after a defined hold time.

#### 2.5.2 Initial State of the System

The initial state is a tensor product:

ρ(0)=|ψ(0)⟩⟨ψ(0)|,with|ψ(0)⟩=|g⟩|1⟩ 14

where represents the initial state where the cavity contains a single-photon Fock state, |1⟩, and the three-level atom is simultaneously in its ground state, |g⟩, poised for the quantum memory protocol to begin.

#### 2.5.3 Lindblad Master Equation for the Open Quantum System

To provide a realistic description of the protocol, the evolution of the system’s density matrix, ρ­(t), must account for its coupling to the environment, which leads to decoherence and dissipation. This evolution is governed by the Lindblad master equation:

$$\frac{d\mathrm{\rho ̂}\left(t\right)}{dt}=-i\left[Ĥ,\mathrm{\rho ̂}\left(t\right)\right]+\underset{k}{\sum }\left({ĉ}_{k}\mathrm{\rho ̂}{ĉ}_{k}^{†}-\frac{1}{2}\left\{{ĉ}_{k}^{†}{ĉ}_{k},\mathrm{\rho ̂}\left(t\right)\right\}\right)$$ 15

The term ${ĉ}_{k}\mathrm{\rho ̂}{ĉ}_{k}^{†}-\frac{1}{2}\left\{{ĉ}_{k}^{†}{ĉ}_{k},\mathrm{\rho ̂}\left(t\right)\right\}$ describes the dissipation for one channel c, where {*Â*, *B̂*} is the anticommutator.^19^

The first channel is for the cavity loss, where the operator ${ĉ}_{1}=\sqrt{\kappa }â$ describes the escape of photons from the cavity with a rate κ.

Next, we include two channels for radiative atomic decay, which is the spontaneous emission of photons from the atom. The operator ${ĉ}_{2}=\sqrt{\Gamma_{e}}{\mathrm{\sigma ̂}}_{ge}$ is for the decay from state |e⟩ to |g⟩ with a rate Γ*_e_*. Also, the operator ${ĉ}_{3}=\sqrt{\Gamma_{f}}{\mathrm{\sigma ̂}}_{ef}$ is for the decay from state |f⟩ to |e⟩ with a rate Γ*_f_*.

The last four channels describe pure dephasing. This process is the loss of phase coherence without loss of population. We include two sources for this. The first source is from environmental phonons, with a rate γ_*phonon*_. The operators ${ĉ}_{4}=\sqrt{\gamma_{phonon}}{\mathrm{\sigma ̂}}_{ee}$ and ${ĉ}_{5}=\sqrt{\gamma_{phonon}}{\mathrm{\sigma ̂}}_{ff}$ describe this dephasing for states |e⟩ and |f⟩. The second source is from material defects, with a rate γ_*defect*_. The final operators, ${ĉ}_{6}=\sqrt{\gamma_{defect}}{\mathrm{\sigma ̂}}_{ee}$ and ${ĉ}_{7}=\sqrt{\gamma_{defect}}{\mathrm{\sigma ̂}}_{ff}$ , are for this defect dephasing and also act on states |e⟩ and |f⟩. For these dephasing channels, the use of projector operators, such as *σ̂*_*ee*_ = |*e*⟩⟨*e*|, as collapse operators in the master equation is the standard method for modeling pure dephasing.

In the reduced basis {|g,1⟩, |e,0⟩, |f,0⟩}, the density operator can be written as,

$$\mathrm{\rho ̂}\left(t\right)=\left(\begin{array}{ccc} \rho_{11} & \rho_{12} & \rho_{13}\\ \rho_{21} & \rho_{22} & \rho_{23}\\ \rho_{31} & \rho_{32} & \rho_{33} \end{array}\right)$$ 16

where each matrix element is defined by,

ρ_11_(*t*) = ⟨*g*,1 | *ρ̂*(*t*) | *g*,1⟩, ρ_12_(*t*) = ⟨*g*,1 | *ρ̂*(*t*) | *e*,0⟩

ρ_13_(*t*) = ⟨*g*,1 | *ρ̂*(*t*) | *f*,0⟩, ρ_21_(*t*) = ⟨*e*,0 | *ρ̂*(*t*) | *g*,1⟩,

ρ_22_(*t*) = ⟨*e*,0 | *ρ̂*(*t*) | *e*,0⟩, ρ_23_(*t*) = ⟨*e*,0 | *ρ̂*(*t*) | *f*,0⟩,

ρ_31_(*t*) = ⟨*f*,0 | *ρ̂*(*t*) | *g*,1⟩, ρ_32_(*t*) = ⟨*f*,0 | *ρ̂*(*t*) | *e*,0⟩,

$$\rho_{33}\left(t\right)=\left\langle f,0\right|\mathrm{\rho ̂}\left(t\right)\left|f,0\right\rangle$$ 17

Equations of motion (simplified from eq 15) define the total pure-dephasing rate γ_*d*_ = γ_*defect*_ + γ_*phonon*_. Then the time derivatives of the nine density-matrix elements read:

dρ11dt=−ig(ρ21−ρ12)−κρ11,dρ22dt=−i[g(ρ12−ρ21)+Ω(t)(ρ32−ρ23)−Γeρ22,dρ33dt=−iΩ(t)(ρ23−ρ32)−Γfρ33,dρ12dt=(iΔe−κ+Γe+γd2)ρ12+iΩ(t)ρ13+ig(ρ11−ρ22),dρ21dt=(−iΔe−κ+Γe+γd2)ρ21−iΩ(t)ρ31−ig(ρ11−ρ22),dρ13dt=(i(Δe+Δf)−κ+Γf+γd2)ρ13+iΩ(t)ρ12+igρ23,dρ31dt=(−i(Δe+Δf)−κ+Γf+γd2)ρ31−iΩ(t)ρ21−igρ32,dρ23dt=(iΔf−Γe+Γf+2γd2)ρ23−iΩ(t)(ρ33−ρ22)+igρ13,dρ32dt=(−iΔf−Γe+Γf+2γd2)ρ32+iΩ(t)(ρ33−ρ22)−igρ31 18

Then the time derivatives of the nine density-matrix elements read as above, and they are ready to be solved using the Runge–Kutta method.

#### 2.5.4 Observables and Metrics

After each time step, we compute three quantities of interest based on the reduced field density matrix ρ̂(t)=Tr[ρ̂(t)] :^19^

Fidelity with respect to the initial single-photon Fock state:

$$F\left(t\right)=\left\langle 1\right|\mathrm{\rho ̂}\left(t\right)\left|1\right\rangle =\rho_{11}\left(t\right)$$ 19

Average photon number:

$$\left\langle n\left(t\right)\right\rangle =Tr\left[\mathrm{\rho ̂}\left(t\right)\left(Î⊗{â}^{†}â\right)\right]=\rho_{11}\left(t\right)$$ 20

Third, the Population of the Storage State. This shows how much of the excitation is correctly transferred and stored in the long-life Rydberg state |f⟩:

$$P\left(t\right)=Tr\left[\mathrm{\rho ̂}\left(t\right)\left({\mathrm{\sigma ̂}}_{ff}⊗Î\right)\right]=\rho_{33}\left(t\right)$$ 21

These quantities are computed over a discretized time range, allowing for a quantitative analysis of the protocol’s performance. The results demonstrate how the engineered light-matter coupling within the cavity QED framework enables the coherent storage and retrieval of a quantum state, even in the presence of realistic decoherence sources, including photonic loss, atomic decay, and pure dephasing.

## 3 Results

### 3.1 Dipole Moment Convergence Analysis

Figure 2b, shows the convergence behavior of the total dipole moment of the METPA crystal along the iterative procedure using ICE approach. At the start of the simulation (iteration zero), the dipole moment, starts around 16.0 D. After some steps, the value increases rapidly and reaches a stable region near 17.65 D from the fourth iteration. This fast convergence, clearly demonstrates the efficiency of the ICE approach for capture polarization effects caused by the crystalline field. The ICE approach appears to show good agreement with experimental results (see refs 9,10,20, and 21 ).

The total dipole moment of 17.65 D is significant higher than that of the isolated molecule, which indicates the important contribution of the crystal packing to the total dipole moment response of the system. Such increase is typical for polar organic systems, where the molecular arrangement in three dimensions, causing increased electronic delocalization and stronger dipole alignment. Furthermore, the small variation observed in the last iterations suggests that the system has reached a reliable stationary configuration, which is suitable for further estimation of nonlinear optical properties including the third-order susceptibility χ^(3)^.

### 3.2 Charge Redistribution in the Crystalline Environment

Table 1 shows the atomic partial charges of the isolated molecule (step 0) and the final charge (step 12, embedded molecule). These values were obtained with the ICE approach, which allows iterative adjustment of atomic charges based on the electrostatic influence of the surrounding embedded molecule.

**Table 1:** Atomic Partial Charges of the METPA Molecule: Isolated (Step 0) vs. Embedded (Step 12) ^a^

number	label	q_initial_ (e)	q_final_ (e)	Δq (e)
1	O4	–0.704	–0.786	–0.082
2	O5	–0.514	–0.495	0.019
3	O6	–0.480	–0.551	–0.071
4	O7	–0.547	–0.475	0.073
5	O8	–0.458	–0.510	–0.051
6	O9	–0.452	–0.501	–0.049
7	O10	–0.455	–0.499	–0.044
8	N4	0.896	0.954	0.058
9	N5	0.847	0.833	–0.014
10	N6	0.718	0.722	0.004
11	C7	0.594	0.612	0.018
12	C8	–0.264	–0.272	–0.009
13	C9	–0.049	–0.031	0.018
14	C10	–0.059	–0.037	0.022
15	C11	–0.039	–0.064	–0.025
16	C12	–0.274	–0.315	–0.041
17	H9	0.163	0.233	0.069
18	H11	0.160	0.212	0.052
19	O1	–0.425	–0.455	–0.030
20	O2	–0.455	–0.521	–0.066
21	O3	–0.730	–0.867	–0.138
22	N1	–0.101	–0.205	–0.104
23	N2	0.031	–0.111	–0.142
24	N3	0.840	0.883	0.043
25	C1	0.216	0.351	0.135
26	C2	–0.141	–0.032	0.108
27	C3	0.015	0.000	–0.015
28	C4	–0.125	–0.007	0.118
29	C5	0.477	0.598	0.121
30	C6	–0.191	–0.307	–0.117
31	H1	0.320	0.373	0.053
32	H3	0.429	0.501	0.073
33	H3A	0.198	0.206	0.008
34	H4A	0.108	0.085	–0.022
35	H4B	0.109	0.090	–0.019
36	H5A	0.022	–0.025	–0.047
37	H5B	–0.050	–0.054	–0.003
38	H6A	0.174	0.200	0.026
39	H6B	0.105	0.154	0.049
40	H6C	0.092	0.115	0.022

a Figure 1 shows the details of the atoms with their labels.

The results show a significant redistribution of electron density across several atoms, evidencing molecular polarization under the crystal field. This redistribution strongly affects macroscopic observables, including the dipole moment and the nonlinear optical response.

Donor Atoms (Δ*q* < 0)

- N2 (Δq = −0.142) and N1 (Δq = −0.104) lost positive charge, which suggests a donor role within hydrogen bonds or extended π-systems.
- O3 (Δq = −0.138) and O4 (Δq = −0.082) also show lower electron density. These oxygen atoms likely participate in intermolecular interactions and hydrogen bonding networks.

**Acceptor Atoms (Δ** *q* **> 0)**

- C1 (Δq = +0.135), C5 (Δq = +0.121), and C4 (Δq = +0.118) gained electron density, indicating charge accumulation possibly driven by conjugation effects and interaction with the picrate moiety.
- N4 (Δq = +0.058) and H9 (Δq = +0.069) also reveal polarization, which may increase local electrostatic interactions and stabilize the crystalline configuration.

These charge transfers are directly related to the improvement of the third-order nonlinear optical susceptibility χ^(3)^. Donor and acceptor sites increase the polarizability of the system and result in higher values of second hyperpolarizability γ, which is a fundamental parameter in predicting χ^(3)^.

Moreover, the difference between the isolated and embedded charge distribution confirms that electronic properties cannot be well described without considering the full crystalline environment.

This analysis confirms that the crystalline surroundings significantly influence the electronic distribution of the METPA molecule, causing non-negligible polarization effects. These modifications are essential for understanding and quantifying the nonlinear optical behavior of the system and validate the use of METPA in advanced photonic and quantum optical applications.

### 3.3 Discussion of Nonlinear Optical Properties

The nonlinear optical behavior of the METPA crystal was evaluated under both static and dynamic cases, see Table 2. These calculations were executed to investigate the material’s third-order nonlinear susceptibility, χ^(3)^, and to assess its potential for photonic applications.

**Table 2:** Static and Dynamic Results for Refractive Index (n), Linear Polarizability (10^–24^ esu), Average Second Hyperpolarizability (10^–36^ esu), and Third-Order Nonlinear Susceptibility (10^–20^(m/V)^2^) of the METPA in Crystalline Phase

electric parameters	static	λ = 532 nm
n(ω)	1.69	1.79
⟨α(−ω; ω)⟩	35.32	39.39
⟨γ(0; 0; 0; 0)⟩	29.68	
⟨γ(−ω; ω; 0; 0)⟩		67.14
⟨γ(−ω; ω; ω; – ω)⟩	35.32	104.6
χ^(3)^(−ω; ω; ω; – ω)	0.72	3.4

In the static case, the refractive index was found around n = 1.69, and the average linear polarizability ⟨α(0; 0)⟩ was estimated at 35.32 × 10^–24^ esu. The computed static third-order susceptibility was χ^(3)^ = 7.2 × 10^–21^ (m/V)^2^, associated with a second hyperpolarizability ⟨γ(0; 0; 0; 0)⟩ of 29.68 × 10^–36^ esu.

These results indicate an intrinsic third-order response, even in the absence of external optical fields. The relatively high χ^(3)^ value under static conditions supports the presence of strong polarizability in the crystal.

When evaluated at a wavelength of 532 nm, the refractive index increased to n = 1.79, and the dynamic average linear polarizability ⟨α­(−ω; ω)⟩ was slightly higher at 39.39 × 10^–24^ esu. The dynamic third-order susceptibility rose substantially to χ^(3)^ = 3.4 × 10^–20^ esu, which is nearly five times the value obtained in the static regime. The calculated hyperpolarizabilities at this wavelength, ⟨γ­(−ω; ω; ω; – ω)⟩ = 104.6 × 10^–30^ esu and ⟨γ­(−ω; ω; 0; 0)⟩ = 67.14 × 10^–36^ esu, suggest that the crystal shows increased nonlinear performance under optical excitation.

This behavior is not linked to a resonant transition but rather to the field-induced redistribution of electronic density, which is supported by our ICE analysis showing marked charge transfer between electronegative oxygen and nitrogen atoms and the electron-rich aromatic carbons. For example, atoms such as O3, O4, and N2 showed significant changes in partial charges (Δq < −0.08), while atoms such as C1, C5, and C4 had Δ*q* > 0.11, indicating internal donor–acceptor polarization that probably contributes to the third-order nonlinear effects.

When compared to other organic nonlinear crystals under the same excitation wavelength, METPA exhibits competitive performance. At λ = 532 nm, METPA’s χ^(3)^ = 3.4 × 10^–20^ (m/V)^2^) surpasses values reported for crystals such as

The METPA crystal presents a good nonlinear optical response, both in the static and dynamic regimes. The increased χ^(3)^ values, supported by clear charge polarization patterns and strong electronic delocalization, indicate that METPA is a suitable candidate for integration into third-order optical devices. The comparison with literature data further confirms its relevance in advancing the field of organic photonic materials, reaching up to 178 times greater than (2E)-1-(3-bromophenyl)-3-[4­(methylsulfanyl)­phenyl]­prop-2-en-1-one^22,23^ as shown in Table 3.

**Table 3:** Third-Order Nonlinear Susceptibility (10^–20^(m/V)^2^) for METPA Compared with the Dynamic Experimental Results for Some Organic Nonlinear Crystals

	**λ** (***nm***)	**χ** ^(**3**)^
METPA (present work)	532	3.40
(2E)-3-(3-methylphenyl)-1-(4-nitrophenyl)prop-2-en-1-one^22^	532	2.77
1-(5-chlorothiophen-2-yl)-3-(2,3-dimethoxyphenyl)prop-2-en-1-one^22,23^ ]	532	0.24
N-(2-hydroxyphenyl)-3-hydroxy-4-iminocyclohexa-2,5-dien-1-one^24^	532	0.15
copper(II) complex Cu^24^	532	0.06
zinc(II) complex Zn^24^	532	0.213
nickel(II) complex Ni^24^	532	0.144
(2E)-3[4(methylsulfanyl)phenyl] −1-(4-nitrophenyl)prop-2-en-1- one^22,23^	800	0.023
(2E)-1-(4-bromophenyl)-3-[4(methylsulfanyl)phenyl]prop-2-en-1-one^22,25^	800	0.023
(2E)-1-(3-bromophenyl)-3-[4(methylsulfanyl)phenyl]prop-2-en-1-one^22,23^	800	0.019

### 3.4 Influence of System Parameters on Protocol Performance

The analysis of the results shows that the performance of the ORCA protocol is critically governed by two things: the cooperativity ratio *g*/κ (which is linked to the cavity quality factor Q) and the selection of the Rydberg state |*f*⟩. For high values of Q = 10^8^ (this means *g*/κ ≈ 980, we can see peak fidelities over 80% (84.51% for |*f*⟩ = |3⟩.and 80.47% for |*f*⟩ = |5⟩. This performance is supported by vacuum Rabi oscillations at 15.6 GHz (see Figures 3(a) and 4(a)), which is typical for strong light-matter coupling. In these conditions, the suppression of cavity loss (κ) preserves the coherence during the storage and retrieval, which follows a STIRAP-like process (**Sti**mulated **R**aman **A**diabatic **P**assage).^26^ However, the final fidelity changes very much with the Rydberg state: while |*f*⟩ = |3⟩ reaches only 34.71% final fidelity, the state |*f*⟩ = |5⟩ achieves 75.60%, which shows it is more robust to decoherence over time.

**Figure 3:**
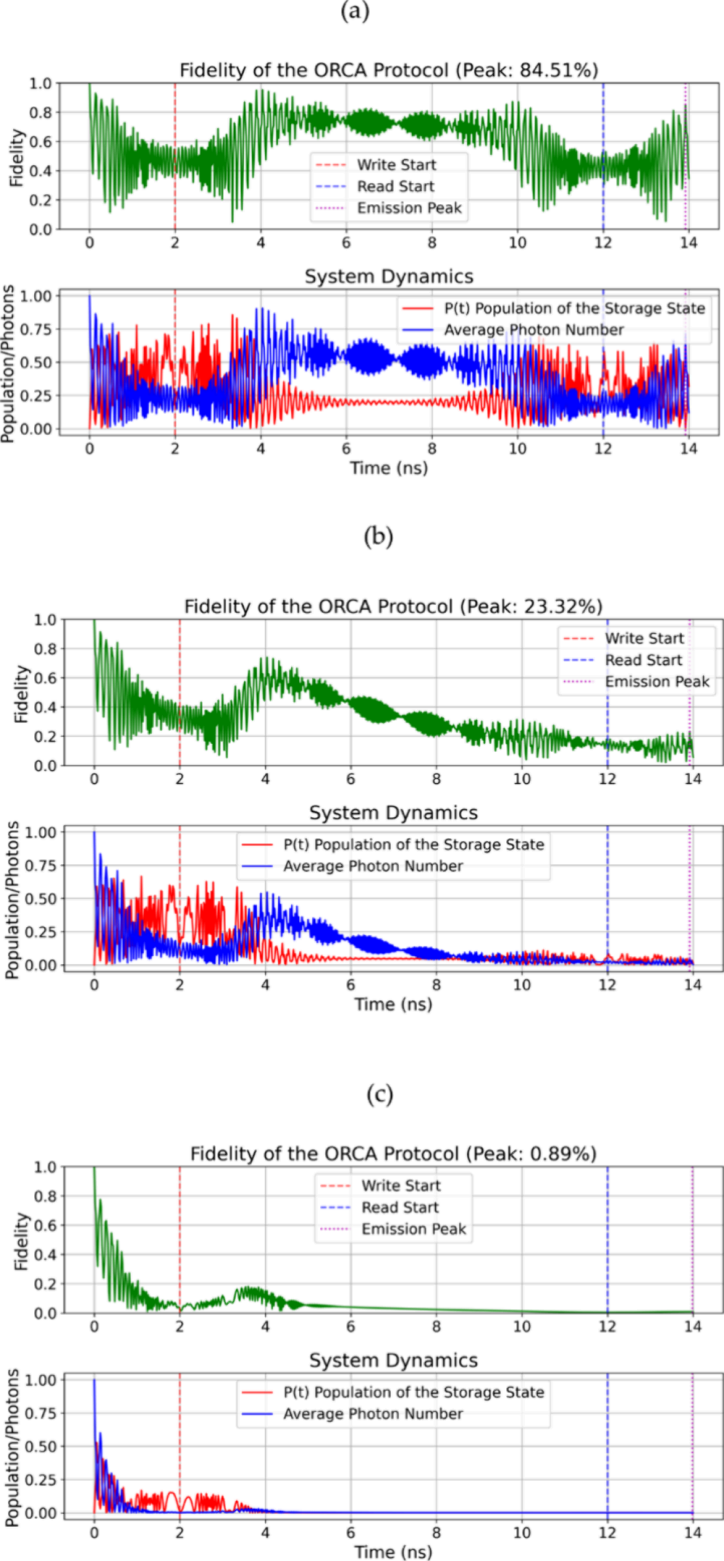
Quantum dynamics of the ORCA protocol. Fidelity of the retrieved photonic state shows oscillations due to coherent STIRAP dynamics and quantum interference during storage and retrieval. Average photon number ⟨n⟩ (blue) and Rydberg state population Pf (red) exhibit vacuum Rabi oscillations at frequency 2g = 15.6 GHz, characteristic of strong light-matter coupling (a) (g/κ ≈ 980) (b) (g/κ ≈ 98), and (c) (g/κ ≈ 9.8). Vertical markers: write pulse start (red), read pulse start (blue), and emission peak (purple). Parameters: Storage State |*f*⟩ = |3⟩, Δe = −20 GHz, Δ*f* = +15 GHz, τ_hold_ = 10 ns, ε0 = 8.854 × 10^–12^ F/m, ℏ = 1.055 × 10^–34^ J·s, and effective volume V = 10^–20^ m^3^.

**Figure 4:**
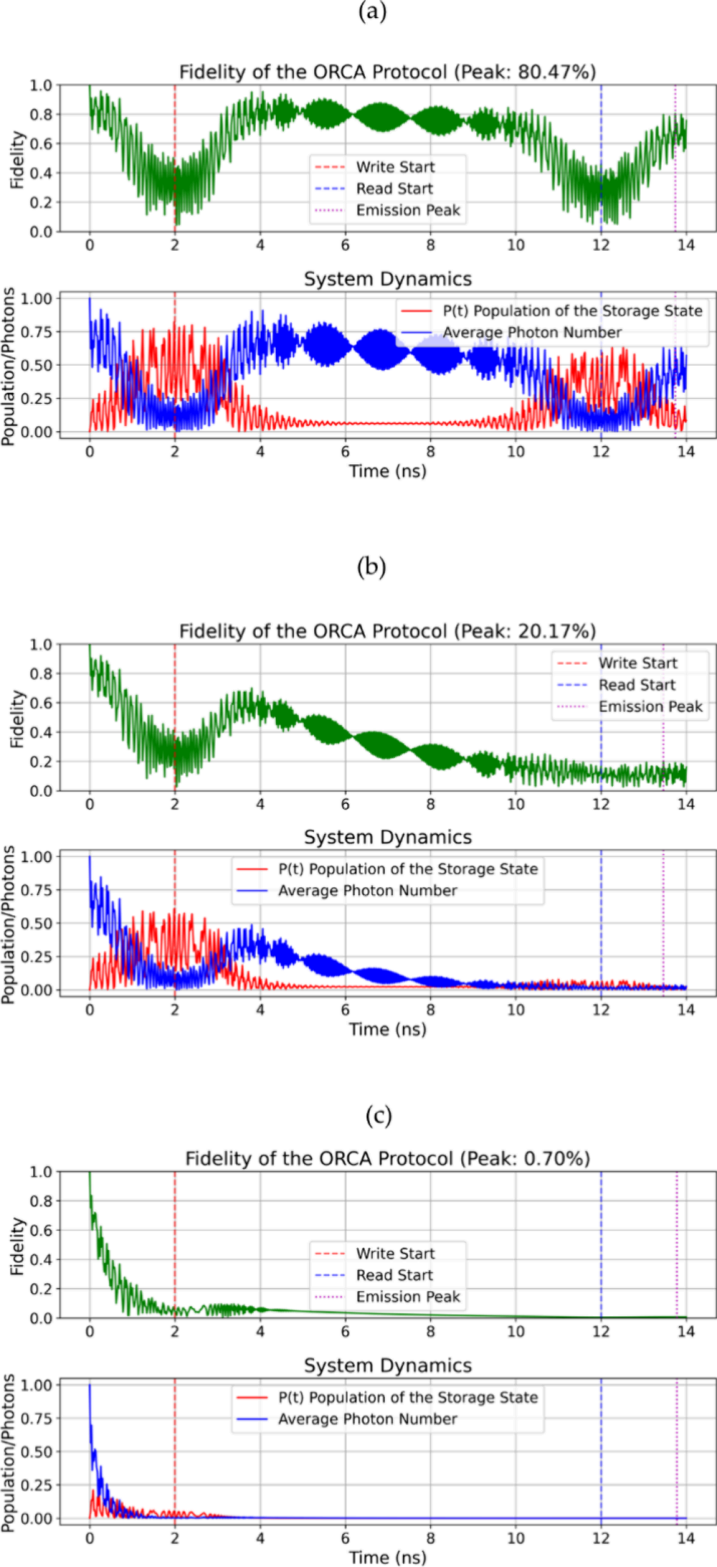
Quantum dynamics of the ORCA protocol. Fidelity of the retrieved photonic state shows oscillations due to coherent STIRAP dynamics and quantum interference during storage and retrieval. Average photon number ⟨n⟩ (blue) and Rydberg state population Pf (red) exhibit vacuum Rabi oscillations at frequency 2g = 15.6 GHz, characteristic of strong light-matter coupling. (a) (g/κ ≈ 980) (b) (g/κ ≈ 98), and (c) (g/κ ≈ 9.8). Parameters: Storage State |*f*⟩ = |5⟩, Δe = −20 GHz, Δ*f* = +15 GHz, τ_hold_ = 10 ns, ε0 = 8.854 × 10*^–12^* F/m, ℏ = 1.055 × 10*^–34^* J·s, and effective volume V = 10^–*20*^ m*^3^*.

When we reduce Q to 10^7^ (*g*/κ ≈ 98) the fidelity degrades very much (for example, a drop from 84.51% to 23.32% at the peak for state |3⟩, Figure 3(b)). This is because the higher cavity loss disturbs the adiabatic transfer process. At Q = 10^6^ (*g*/κ ≈ 9.8), the protocol collapses, with fidelities below 1% (0.89% for |3⟩, Figure 3(c)). This shows that κ dominates over the coupling *g*, which destroys the coherence. It is interesting to note that higher Rydberg states (like |5⟩) help to mitigate this effect. This is because their intrinsic loss rate (Γ*_f_*) is 50% smaller (3 kHz vs 6 kHz for |3⟩), even with a reduced dipole moment μ_*ef*_ (0.63 D vs 1.15 D).

The time dynamics (Figures 3 and 4) reveal oscillations in the fidelity. This is from quantum interference during the STIRAP process, and the amplitude of these oscillations decreases as *g*/κ decreases. The synchronization between the write pulse (red marker), read pulse (blue), and the peak of emission (purple) is very important. It helps to maximize the population in |*f*⟩ (up to 85.57% with high Q) and the efficiency of retrieval. Parameters like the effective volume (V = 10^–20^ m^3^) and the control intensity (10^11^ W/m^2^) make sure the conditions are stable for Rydberg polaritons, with the Rabi drive Ω_0_ being much larger than the detunings (Δ_e_=–20 GHz, Δ_f_=+15 GHz). Table 4 summarizes the parameters used for the simulations presented in Figures 3 and 4.

**Table 4:** Comparative Analysis of Parameters and Results for the Simulated Scenarios

parameter	unit	Figure 3 (a)	Figure 3 (b)	Figure 3 (c)	Figure 4 (a)	[Fig fig4] Figure 4 (b)	[Fig fig4] Figure 4 (c)
storage state | ***f*** ⟩		|3⟩	|3⟩	|3⟩	|5⟩	|5⟩	|5⟩
quality factor, Q		10^8^	10^7^	10^6^	10^8^	10^7^	10^6^
**μ**_*ge*_	(D)	0.90	0.90	0.90	0.90	0.90	0.90
**μ**_*ef*_	(D)	1.15	1.15	1.15	0.63	0.63	0.63
control intensity	W/m^2^	10^11^	10^11^	10^11^	10^11^	10^11^	10^11^
g/2π	GHz	7.80	7.80	7.80	7.80	7.80	7.80
Ω_0_/2π	GHz	50.3	50.3	50.3	27.4	27.4	27.4
cavity loss, κ/2π	MHz	7.94	79.4	794	7.94	79.4	794
**Γ**_*e*_/2π	kHz	750	750	750	750	750	750
**Γ**_*f*_/2π	kHz	6	6	6	3	3	3
**γ**_*phonon*_/2π	kHz	160	160	160	160	160	160
**γ**_*defect*_/2π	kHz	160	160	160	160	160	160
peak fidelity	%	84.51	23.32	0.89	80.47	20.17	0.70
final fidelity	%	34.7	5.59	0.88	75.6	16.1	0.69
max. population in P(t)	%	85.5	66.7	52.7	82.3	60.7	21.1

The conditions for an efficient ORCA operation are therefore a system with a cooperativity ratio *g*/κ > 98, and the selection of a Rydberg storage state^27,28^ with a principal quantum number *n* ≥ 5 to minimize its intrinsic decay rate, Γ*_f_*. This balance allows final fidelities over 75%, which positions the protocol as a viable candidate for quantum memories based on Rydberg-cavity systems.

## 4 Conclusion

We have demonstrated that the crystalline environment considerably increases the nonlinear optical response of the METPA molecule. By utilizing the crystalline environment through the ICE approach, we have confirmed that METPA exhibits a substantially increased dipole moment and third-order nonlinear optical response.

The computed third-order susceptibility (χ^(3)^) of approximately 3.4 × 10^–20^ (m/V)^2^) at 532 nm is higher than values reported for several organic crystals, underscoring its robust polarizability and potential for advanced photonic devices. Furthermore, quantum memory simulations for the ORCA protocol were performed, modeled via a Lindblad master equation that quantifies fidelity, photon number, and state populations for an initial single-photon Fock state.

The results revealed that the protocol’s performance is critically dependent on the cavity quality factor, Q, which dictates the cooperativity ratio g/κ. In the strong coupling regime (g/κ ≈ 980), achieved with a high-Q cavity (Q = 10^8^), a peak retrieval fidelity of 84.51% was observed. Conversely, the fidelity collapses to less than 1% when the system leaves the strong-coupling regime (g/κ ≈ 9.8) for a low-Q cavity (Q = 10^6^).

These results emphasize the importance of the intrinsic electronic properties of the METPA crystal, whose strong transition dipole moments (e.g., μ_ge_ and μ_ef_) enable the large atom-cavity (g) and control-field (Ω_0_) coupling rates required for an efficient protocol. This demonstrates that METPA, with its robust electronic structure; also responsible for its strong third-order nonlinear response; stands out as a promising candidate for next-generation quantum information protocols when integrated into an optimized, low-loss cavity QED platform.

## Supplementary Material



## Notes

Table S1 – Energy levels and atomic state descriptions; Table S2 – Electric dipole moments for atomic transitions (in a.u.); Explanation of energy level indexing and example calculations; Formulas for calculating detuning and coupling strengths in Off-Resonant Cascaded Absorption protocol (PDF)

## References

[1] Heshami K., England D. G., Humphreys P. C., Bustard P. J., Acosta V. M., Nunn J., Sussman B. J. (2016). Quantum
Memories: Emerging Applications
and Recent Advances. J. Mod. Opt.

[2] Lvovsky A. I., Sanders B. C., Tittel W. (2009). Optical Quantum Memory. Nat. Photonics.

[3] Serrano D., Kuppusamy S. K., Heinrich B., Fuhr O., Hunger D., Ruben M., Goldner P. (2022). Ultra-Narrow Optical Linewidths in
Rare-Earth Molecular Crystals. Nature.

[4] de
Riedmatten H., Afzelius M., Staudt M. U., Simon C., Gisin N. (2008). A Solid-State Light–Matter Interface at the Single-Photon
Level. Nature.

[5] White T., Schoof T., Yakubov S., Tolstikova A., Middendorf P., Karnevskiy M., Mariani V., Henkel A., Klopprogge B., Hannappel J., Oberthuer D., De Gennaro Aquino I., Egorov D., Munke A., Sprenger J., Pompidor G., Taberman H., Gruzinov A., Meyer J., Hakanpää J., Gasthuber M. (2025). Real-Time
Data Processing for Serial Crystallography Experiments. IUCrJ..

[6] Popczyk A., Aamoum A., Migalska-Zalas A., Płóciennik P., Zawadzka A., Mysliwiec J., Sahraoui B. (2019). Selected Organometallic
Compounds for Third Order Nonlinear Optical Application. Nanomaterials.

[7] Swamynayaka A., Venkatesha K., Harish K. K., Revanna B. N., Venkatesh C., Madegowda M., Hegde T. A. (2023). Third-Order Nonlinear Response of
a Novel Organic Acetohydrazide Derivative: Experimental and Theoretical
Approach. Opt Mater. (Amst).

[8] Ajibola A. A., Karthick T., Roshni J., Sieroń L., Maniukiewicz W. (2025). Metronidazole-Picric Acid Salt Formation:
Synthesis,
Characterization, Hirshfeld Surface Analysis, Single Crystal X-Ray
Diffraction, Computational Studies, and Molecular Docking Studies
against Clostridioides Difficile. J. Mol. Struct..

[9] Valverde C., Osório F. A. P., Fonseca T. L., Baseia B. (2018). DFT Study of Third-Order
Nonlinear Susceptibility of a Chalcone Crystal. Chem. Phys. Lett..

[10] Barbosa M. R., Costa I. S. D., Lopes T. O., Valverde C., Machado D. F. S., Oliveira H. C. B. D. (2022). Theoretical Model of Polarization
Effects on Third-Order NLO Properties of the Stilbazolium Derivative
Crystal. J. Phys. Chem. A.

[11] Valverde C., Medeiros R., Franco L. R., Osório F. A. P., Castro M. A., Fonseca T. L. (2023). Theoretical Investigation
on the
Linear and Nonlinear Optical Properties of DAPSH Crystal. Sci. Rep..

[12] Valverde C., Osório F. A. P. (2024). Study
of Nonlinear Optical Properties of a Self-Healing
Organic Crystal. ACS Omega.

[13] Deptuch A., Sęk B., Lalik S., Zając W., Ossowska-Chruściel M. D., Chruściel J., Marzec M. (2024). Structural Study of Nematogenic Compound
5OS5. Crystals (Basel).

[14] Srivathsan, B. ; Gartman, R. ; Francis-Jones, R. J. A. ; Mosley, P. ; Nunn, J. Scalable Linear-Cavity Enhanced Quantum Memory. arXiv 2025. 10.48550/arXiv.2503.14212. 41138123

[15] Kaczmarek K. T., Ledingham P. M., Brecht B., Thomas S. E., Thekkadath G. S., Lazo-Arjona O., Munns J. H. D., Poem E., Feizpour A., Saunders D. J., Nunn J., Walmsley I. A. (2018). High-Speed Noise-Free
Optical Quantum Memory. Phys. Rev. A (Coll Park).

[16] Poojith N., Rani N. U., Potla K. M., Rose J. J., Suchetan P. A., Pillai R. R., Vankayalapati S. (2021). An Analysis of Structural, Spectroscopic,
Quantum Chemical and in Silico Studies of Ethyl 3-[(Pyridin-2-Yl)­Amino]­Propanoate:
A Potential Thrombin Inhibitor. J. Mol. Struct..

[17] Murthy P. K., Sheena Mary Y., Shyma Mary Y., Panicker C. Y., Suneetha V., Armaković S., Armaković S. J., Van Alsenoy C., Suchetan P. A. (2017). Synthesis, Crystal
Structure Analysis, Spectral Investigations,
DFT Computations and Molecular Dynamics and Docking Study of 4-Benzyl-5-Oxomorpholine-3-Carbamide,
a Potential Bioactive Agent. J. Mol. Struct..

[18] Frisch, M. J. ; Trucks, G. W. ; Schlegel, H. B. ; Scuseria, G. E. ; Robb, M. A. ; Cheeseman, J. R. ; Scalmani, G. ; Barone, V. ; Petersson, G. A. ; Nakatsuji, H. ; Li, X. ; Caricato, M. ; Marenich, A. V ; Bloino, J. ; Janesko, B. G. ; Gomperts, R. ; Mennucci, B. ; Hratchian, H. P. ; Ortiz, J. V ; Izmaylov, A. F. ; Sonnenberg, J. L. ; Williams-Young, D. ; Ding, F. ; Lipparini, F. ; Egidi, F. ; Goings, J. ; Peng, B. ; Petrone, A. ; Henderson, T. ; Ranasinghe, D. ; Zakrzewski, V. G. ; Gao, J. ; Rega, N. ; Zheng, G. ; Liang, W. ; Hada, M. ; Ehara, M. ; Toyota, K. ; Fukuda, R. ; Hasegawa, J. ; Ishida, M. ; Nakajima, T. ; Honda, Y. ; Kitao, O. ; Nakai, H. ; Vreven, T. ; Throssell, K. ; Montgomery, J. A., Jr. ; Peralta, J. E. ; Ogliaro, F. ; Bearpark, M. J. ; Heyd, J. J. ; Brothers, E. N. ; Kudin, K. N. ; Staroverov, V. N. ; Keith, T. A. ; Kobayashi, R. ; Normand, J. ; Raghavachari, K. ; Rendell, A. P. ; Burant, J. C. ; Iyengar, S. S. ; Tomasi, J. ; Cossi, M. ; Millam, J. M. ; Klene, M. ; Adamo, C. ; Cammi, R. ; Ochterski, J. W. ; Martin, R. L. ; Morokuma, K. ; Farkas, O. ; Foresman, J. B. ; Fox, D. J. Gaussian 16 Revision C.01 2024. Gaussian, Inc.: Wallingford CT, 2016.

[19] Louisell, W. H. Quantum Statistical Properties of Radiation; Wiley series in pure and applied optics; Wiley: New York, NY, 1973.

[20] Fonseca T. L., Sabino J. R., Castro M. A., Georg H. C. (2010). A theoretical investigation
of electric properties of L-arginine phosphate monohydrate including
environment polarization effects. J. Chem. Phys..

[21] Santos O. L., Fonseca T. L., Sabino J. R., Georg H. C., Castro M. A. (2015). Polarization
Effects on the Electric Properties of Urea and Thiourea Molecules
in Solid Phase. J. Chem. Phys..

[22] Prabhu S. R., Jayarama A., Chandrasekharan K., Upadhyaya V., Ng S. W. (2017). Synthesis, Growth, Structural Characterization,
Hirshfeld Analysis
and Nonlinear Optical Studies of a Methyl Substituted Chalcone. J. Mol. Struct..

[23] Prabhu A. N., Upadhyaya V., Jayarama A., Subrahmanya Bhat K. (2013). Synthesis,
Growth and Characterization of π Conjugated Organic Nonlinear
Optical Chalcone Derivative. Mater. Chem. Phys..

[24] Ananthi N., Balakrishnan U., Velmathi S., Manjunath K. B., Umesh G. (2012). Synthesis, Characterization and Third Order Non Linear Optical Properties
of Metallo Organic Chromophores. Optics and
Photonics Journal.

[25] D’silva E. D., Podagatlapalli G. K., Venugopal Rao S., Dharmaprakash S. M. (2012). Study on
Third-Order Nonlinear Optical Properties of 4-Methylsulfanyl Chalcone
Derivatives Using Picosecond Pulses. Mater.
Res. Bull..

[26] Vitanov N. V., Fleischhauer M., Shore B. W., Bergmann K. (2001). Coherent Manipulation
of Atoms Molecules By Sequential Laser Pulses. Adv. At., Mol., Opt. Phys..

[27] Aoki T., Dayan B., Wilcut E., Bowen W. P., Parkins A. S., Kippenberg T. J., Vahala K. J., Kimble H. J. (2006). Observation of Strong
Coupling between One Atom and a Monolithic Microresonator. Nature.

[28] Mabuchi H., Doherty A. C. (2002). Cavity Quantum Electrodynamics:
Coherence in Context. Science (1979).

