# Supplementary material for: A Theoretical Investigation of Third-Order Optical Susceptibility in Metronidazolium-Picrate Crystal and Its Potential for Quantum Memory Applications
Source: ACS Omega. 2025 Aug 21;10(34):38731–9. doi: 10.1021/acsomega.5c03825 (PMC12409683; doi:10.1021/acsomega.5c03825)
Supplement: Supplementary file 1 [file ao5c03825_si_001.pdf]

A Theoretical Investigation of Third-Order Optical Susceptibility in Metronidazolium-Picrate Crystal and Its Potential for Quantum Memory Applications

Clodoaldo Valverde[1,2]

[1] Laboratório de Modelagem Molecular Aplicada e Simulação (LaMMAS), Universidade Estadual de Goiás, 75001-970, Anápolis, GO, Brasil
[2].Universidade Paulista – UNIP, 74845-090, Goiânia, GO, Brasil
Email: valverde@ueg.br

A B S T R A C T

In this work, we report a theoretical investigation of the third-order nonlinear optical properties of the metronidazolium-picrate salt. The effects of the crystal environment are accounted for by the Iterative Charge Embedding approach, and the electronic calculations are carried out at the DFT (CAM-B3LYP/6-311++G(d,p)) level. Furthermore, we use the ab initio results to parameterize a cavity Quantum Electrodynamics model for a quantum memory based on the Off-Resonant Cascaded Absorption protocol. The system's performance is then simulated via a Lindblad-type master equation that includes realistic decoherence channels. Our results confirm a strong third-order susceptibility ($\chi^{(3)}$) of 3.4×10^{-20} $(m/V)^2$ at 532 nm, driven by significant charge polarization in the crystal. The quantum memory simulations, initiated with a single-photon Fock state, reveal that protocol fidelity is critically dependent on the cavity quality factor. A peak retrieval fidelity of 84.51% is achieved in the strong coupling regime, which collapses to less than 1% when the system leaves this regime. These findings demonstrate that METPA is a promising material for quantum photonics, where its strong intrinsic electronic properties can be harnessed in engineered cavity Quantum Electrodynamics systems to realize high-fidelity quantum information protocols.

Table S1 is organized so that each Level Index (0, 1, 2, …) appears in the first column, and its corresponding Energy (eV) in the second.

Table S1: Atomic Energy Levels: Level Index and Corresponding State

Level Index │ Atomic State Description	Energy (eV)
0 – ground state	0.000000
1 – first excited state	3.284500
2 – second excited state	3.498100
3 – third excited state	3.831000
4 – fourth excited state	3.901900
5 – fifth excited state	3.917000
6 – sixth excited state	3.957900
7 – seventh excited state	4.134100
8 – eighth excited state	4.202900
9 – ninth excited state	4.288400

10 – tenth excited state	4.315500
11 – eleventh excited state	4.487400
12 – twelfth excited state	4.506900
13 – thirteenth excited state	4.539500
14 – fourteenth excited state	4.860500
15 – fifteenth excited state	4.926000
16 – sixteenth excited state	4.989800
17 – seventeenth excited state	5.000000
18 – eighteenth excited state	5.068100
19 – nineteenth excited state	5.287700
20 – twentieth excited state	5.397900

To pick out the energy for a given storage level f, follow these steps:

- Locate the Level Index you want (the number you pass as f).
- Find the row whose Level Index matches that number.
- Read the Energy in the same row.

Example 1: f=|3>

- Level Index = 0 → Row 1 → Energy = 0.0000 eV → defines E_g, the ground state |g⟩.
- Level Index = 1 → Row 2 → Energy = 3.2845 eV → defines E_e, the intermediate state |e⟩.
- Level Index = 3 → Row 4 → Energy = 3.8310 eV → defines E_f for f=3, the storage state |f⟩.

Thus for f=3, the storage-state energy **is 3.8310 eV.**

With this value of E_f = 3.8310 eV, E_e = 3.2845 eV and E_g = 0 eV

The detunings Δ_e and Δ_f are simply the offsets of your laser frequencies from the atom's natural transition frequencies:

$$\omega_{ge} = \frac{E_e - E_g}{\hbar}, \qquad \text{(S1)}$$

Is the angular frequency of the |g> → |e> transition.

$$\omega_{ef} = \frac{E_f - E_e}{\hbar}, \qquad \text{(S2)}$$

Is the angular frequency of the |e> → |f> transition.

Detuning definitions

$$\Delta_e = \omega_{signal} - \omega_{ge},$$
$$\Delta_f = \omega_{control} - \omega_{ef} \qquad \text{(S3)}$$

In our setup, the Table 2 provides the electric-dipole moments for every possible transition between the atomic levels. It is arranged as a square matrix whose rows and columns are labeled by the same level indices you use for g, e and f. Each entry μ_{ij} gives the dipole moment for the transition |i⟩→|j⟩ in atomic units.

Example: extracting μ_{ge} and μ_{ef}.

μ_{ge} (ground →intermediate)

Row 0, Column 1 →0.899(a.u.), see Table 2.

- For f = |3>

μ_{ef} is the dipole moment for the |e⟩→|f⟩ transition, i.e.

Row 1, Column 3 → 1.152 a.u.

What you do with these numbers

- **Convert** from atomic units to SI (C·m) by multiplying with your conversion factor.
- **Compute** the coupling strengths:

$$g = \frac{\mu_{ge}}{\hbar}\sqrt{\frac{\hbar\omega_{ge}}{2\epsilon_0 V_{cav}}}, \qquad \text{(S4)}$$

and

$$\Omega_0(t) = \frac{\mu_{ef} E_0}{\hbar}, \qquad \text{(S5)}$$

Example 2: f=|5>

- **Level Index = 5 → Row 6** → Energy = 3.9170 eV → defines E_f for f=5, a different storage state |f⟩.

Thus for f=5, the storage-state energy is **3.9170 eV**.

In each case, you simply match the chosen f index to the Level Index column in Table 1 and read off the energy in that same row.

- For f = |5>, see Table 2.

μ_{ef} is the dipole moment for the |e⟩→|f⟩ transition, i.e.

Row 1, Column 5 → 0.628 a.u.

Table S2: Electric Dipole Moments for Atomic Transitions (in Atomic Units)

	0	1	2	3	4	5	6	7	8	9	10	11	12	13	14	15	16	17	18	19	20
**0**	16.09	0.90	5.50	1.88	0.07	1.36	0.17	0.98	1.81	6.54	0.60	1.59	0.10	0.49	1.30	0.46	0.34	2.86	1.06	2.85	0.76
**1**	0.90	20.75	6.63	1.15	0.01	0.63	0.06	0.23	0.24	0.52	0.45	0.43	0.05	0.02	0.02	2.08	3.29	0.64	0.09	0.46	0.68
**2**	5.50	6.63	13.04	3.50	0.03	2.25	0.01	0.86	1.54	3.83	4.12	4.51	0.01	0.12	0.08	0.84	0.59	0.12	0.55	1.05	0.13
**3**	1.88	1.15	3.50	11.03	0.04	2.14	0.00	0.66	0.16	1.35	1.27	2.62	0.00	0.18	0.23	0.47	1.26	0.00	1.16	1.26	0.35
**4**	0.07	0.01	0.03	0.04	9.75	1.19	0.00	0.37	0.22	0.11	0.26	0.03	0.00	1.52	1.13	0.01	0.01	0.00	0.28	0.06	0.00
**5**	1.36	0.63	2.25	2.14	1.19	15.89	0.02	0.97	4.85	2.39	1.83	3.30	0.04	1.37	2.45	0.16	0.53	0.16	3.44	4.26	0.83
**6**	0.17	0.06	0.01	0.00	0.00	0.02	19.28	0.06	0.09	0.00	0.02	0.00	3.73	0.00	0.00	0.03	0.01	1.47	0.00	0.00	0.15
**7**	0.98	0.23	0.86	0.66	0.37	0.97	0.06	12.14	9.35	2.92	2.85	0.97	0.13	0.58	1.58	0.17	1.12	0.46	3.53	1.08	1.49
**8**	1.81	0.24	1.54	0.16	0.22	4.85	0.09	9.35	10.41	4.25	4.83	1.13	0.22	0.37	1.26	0.89	0.64	0.74	4.19	0.51	2.45
**9**	6.54	0.52	3.83	1.35	0.11	2.39	0.00	2.92	4.25	22.41	2.18	1.61	0.00	0.48	0.30	1.72	1.12	0.00	2.36	0.49	0.00
**10**	0.60	0.45	4.12	1.27	0.26	1.83	0.02	2.85	4.83	2.18	11.26	0.73	0.04	0.45	3.55	0.48	0.92	0.15	2.15	1.51	0.71
**11**	1.59	0.43	4.51	2.62	0.03	3.30	0.00	0.97	1.13	1.61	0.73	11.91	0.00	0.31	1.58	0.80	1.03	0.00	1.58	2.34	0.10
**12**	0.10	0.05	0.01	0.00	0.00	0.04	3.73	0.13	0.22	0.00	0.04	0.00	18.32	0.00	0.00	0.02	0.01	0.20	0.00	0.00	0.04
**13**	0.49	0.02	0.12	0.18	1.52	1.37	0.00	0.58	0.37	0.48	0.45	0.31	0.00	19.24	2.17	0.03	0.04	0.00	0.41	0.50	0.07
**14**	1.30	0.02	0.08	0.23	1.13	2.45	0.00	1.58	1.26	0.30	3.55	1.58	0.00	2.17	20.54	0.00	0.06	0.08	2.66	0.72	0.30
**15**	0.46	2.08	0.84	0.47	0.01	0.16	0.03	0.17	0.89	1.72	0.48	0.80	0.02	0.03	0.00	21.68	3.68	0.68	0.00	0.94	7.73
**16**	0.34	3.29	0.59	1.26	0.01	0.53	0.01	1.12	0.64	1.12	0.92	1.03	0.01	0.04	0.06	3.68	11.57	0.17	0.06	0.25	1.95
**17**	2.86	0.64	0.12	0.00	0.00	0.16	1.47	0.46	0.74	0.00	0.15	0.00	0.20	0.00	0.08	0.68	0.17	14.83	0.01	0.00	2.48
**18**	1.06	0.09	0.55	1.16	0.28	3.44	0.00	3.53	4.19	2.36	2.15	1.58	0.00	0.41	2.66	0.00	0.06	0.01	17.75	0.89	0.32
**19**	2.85	0.46	1.05	1.26	0.06	4.26	0.00	1.08	0.51	0.49	1.51	2.34	0.00	0.50	0.72	0.94	0.25	0.00	0.89	15.42	0.27

**20**	0.76	0.68	0.13	0.35	0.00	0.83	0.15	1.49	2.45	0.00	0.71	0.10	0.04	0.07	0.30	7.73	1.95	2.48	0.32	0.27	17.12
